# Let distortion-guided restoration: a physics-informed learning framework to correct prostate diffusion MRI artifacts

**DOI:** 10.1093/radadv/umag031

**Published:** 2026-07-14

**Authors:** Ziyang Long, Nader Binesh, Lixia Wang, Archana Vadiraj Malaji, Chia-Chi Yang, Haoran Sun, Rola Saouaf, Timothy Daskivich, Hyung Kim, Yibin Xie, Debiao Li, Hsin-Jung Yang

**Affiliations:** Biomedical Imaging Research Institute, Cedars-Sinai Medical Center, Los Angeles, CA 90048, United States; Department of Bioengineering, University of California, Los Angeles, Los Angeles, CA, 90095, United States; Department of Imaging, Cedars-Sinai Medical Center, Los Angeles, CA, 90048, USA; Biomedical Imaging Research Institute, Cedars-Sinai Medical Center, Los Angeles, CA 90048, United States; Biomedical Imaging Research Institute, Cedars-Sinai Medical Center, Los Angeles, CA 90048, United States; Biomedical Imaging Research Institute, Cedars-Sinai Medical Center, Los Angeles, CA 90048, United States; Biomedical Imaging Research Institute, Cedars-Sinai Medical Center, Los Angeles, CA 90048, United States; Department of Bioengineering, University of California, Los Angeles, Los Angeles, CA, 90095, United States; Department of Imaging, Cedars-Sinai Medical Center, Los Angeles, CA, 90048, USA; Urology, Cedars-Sinai Medical Center, Los Angeles, CA, 90048, United States; Urology, Cedars-Sinai Medical Center, Los Angeles, CA, 90048, United States; Biomedical Imaging Research Institute, Cedars-Sinai Medical Center, Los Angeles, CA 90048, United States; Biomedical Imaging Research Institute, Cedars-Sinai Medical Center, Los Angeles, CA 90048, United States; Department of Bioengineering, University of California, Los Angeles, Los Angeles, CA, 90095, United States; Biomedical Imaging Research Institute, Cedars-Sinai Medical Center, Los Angeles, CA 90048, United States

**Keywords:** prostate MRI, diffusion-weighted imaging, distortion correction

## Abstract

**Background:**

Susceptibility-induced distortion in single-shot echo-planar imaging (ssEPI) prostate diffusion-weighted imaging (DWI) can degrade lesion conspicuity, apparent diffusion coefficient (ADC) estimation, and confidence in prostate MRI interpretation. Conventional correction strategies typically require additional acquisitions and may be limited in severe artifact settings.

**Purpose:**

To develop a physics-informed deep learning framework, distortion-guided restoration (DGR), for acquisition-free correction of ssEPI distortions in prostate DWI.

**Materials and methods:**

Distortion-guided restoration was proposed to learn the inverse of a physically simulated ssEPI distortion process. Clinical prostate DWI and T_2_-weighted (T_2_W) images from 408 3T MR examinations originating from 2 clinical datasets, acquired on Siemens 3T MAGNETOM Vida and Biograph mMR scanners, were used for paired distorted/undistorted training data across low-b DWI (*b* = 50 s/mm^2^), and scanner-provided ADC maps. The restoration network integrates a convolutional neural network (CNN)-based geometric correction module with a conditional diffusion refinement module, both guided by co-registered T_2_W images. Model selection and benchmarking were performed on a held-out synthetic test set (*n* = 34), against conventional algorithms using correction field mapping (FSL FUGUE and TOPUP). Model performance was evaluated in a retrospective study on 34 clinical scans with severe susceptibility artifacts drawn from the same clinical datasets, using quantitative image metrics and blinded radiologist scoring.

**Results:**

On synthetic data, the proposed DGR model achieved the highest peak signal-to-noise ratio (PSNR) and lowest normalized mean squared error (NMSE) across low-b DWI (0.089; 95% CI, 0.072-0.105) and ADC maps (0.062; 95% CI, 0.053-0.072), outperforming FSL TOPUP and FUGUE (all *P* < .001). In the 34 clinical cases affected by susceptibility artifacts, including 16 cases with histologically confirmed lesions (16/34, 47.1%), DGR substantially improved geometric fidelity (2.74 to 3.29), identified all histologically confirmed lesions (16/16, 100%), and enhanced overall image quality and diagnostic confidence (all *P* < .001).

**Conclusion:**

This proof-of-concept study suggests that a physics-informed hybrid CNN-diffusion framework offers a practical and acquisition-free solution for correcting severe prostate DWI distortions. It warrants further development for clinical utility and implementation.

Summary A physics-informed convolutional neural network (CNN)-diffusion framework enables correction of susceptibility-induced artifacts in prostate diffusion MRI without additional data acquisition by learning the inverse of a forward distortion process.

**Key Results** 

- Distortion-guided restoration (DGR) outperformed conventional B_0_ correcting methods (eg, FUGUE and TOPUP) on synthetic data, achieving higher peak signal-to-noise ratio (PSNR) and lower normalized mean squared error (NMSE) on low-b diffusion-weighted imaging (DWI) and ADC (all *P* < .001).
- In a clinical cohort, DGR improved geometric fidelity 2.7→3.3, image quality 2.7→2.9, and diagnostic confidence 2.7→3.1 on a 5-point Likert scale (all *P* < .05).
- In histopathology-confirmed cases, DGR preserved true lesions with no false positives.

## Introduction

Multiparametric MRI (mpMRI), combining T_2_-weighted (T_2_W) imaging with multi-b-value diffusion-weighted imaging (DWI), is central to prostate cancer detection, PI-RADS grading, and treatment planning.^1^^,^^2^ High-b DWI and the derived apparent diffusion coefficient (ADC) maps provide key biomarkers of lesion conspicuity and tumor aggressiveness, yet they are intrinsically low in signal-to-noise ratio (SNR) and susceptible to imaging artifacts. Even moderate distortions can blur lesion boundaries, shift anatomical landmarks, or corrupt ADC estimates, which directly impact diagnostic confidence and the accuracy of MR-guided biopsy targeting.^3^^,^^4^ As a result, susceptibility-induced distortion has become one of the most persistent technical barriers to high-quality prostate mpMRI.

These challenges are greatly amplified in patients with bowel distention and metallic implants (eg, unilateral or bilateral hip prostheses).^5^^,^^6^ Susceptibility perturbations from metal extend into the pelvis, generating B_0_ gradients far exceeding the effective EPI bandwidth. As a result, single-shot echo-planar imaging (ssEPI) (the clinical standard for prostate DWI) images are susceptible to extreme stretching, compression, pixel pile-up, and complete signal dropout within the prostate gland. While specialized hardware solutions like local shim coils have shown promise in mitigating these artifacts, they require dedicated equipment, which is not universally available.^7^ Consequently, with hip arthroplasty increasingly common in the same population at highest risk for prostate cancer, robust distortion correction using standard clinical hardware remains an urgent and unresolved clinical need.^8^

Classical correction techniques require an additional input of the B_0_ field map (eg, FMRIB’s Utility for Geometrically Unwarping EPI [FUGUE], part of the FMRIB Software Library [FSL]) and reverse phase-encoding (PE) reconstruction (eg, Tool for Estimating and Correcting Susceptibility-Induced Distortions [TOPUP]).^9–12^ These methods can perform adequately when distortion is mild, and the underlying signal remains intact. However, all rely on accurate estimation of pixel displacement or local image gradients of the B_0_ maps. Near metal implants or when movement is present, these assumptions break down. Severe signal dropout invalidates the forward signal model, large B_0_ gradients invalidate linear approximations, and registration becomes ill-posed when anatomical structures are stretched or collapsed beyond recovery.^13^ Moreover, additional acquisitions such as dual PE images or reliable B_0_ maps are often impractical in routine prostate MRI and may themselves fail in regions of severe susceptibility.

More recently, deep learning approaches have been explored to improve diffusion-weighted prostate imaging and mitigate metal-related artifacts. Vendor-specific reconstruction networks and post-processing models can enhance SNR and perceived image quality, and physics-informed or attention-guided networks have shown promise for multi-shot DWI reconstruction or metal artifact reduction.^14–17^ Yet these methods typically target denoising or generic artifact suppression, require specialized acquisitions (eg, multi-shot, dual-polarity readouts), or address different anatomical sites and artifact mechanisms.^18^^,^^19^ None directly learns to undo the extreme geometric warping of ssEPI prostate DWI caused by hip prostheses or rectal air distention using only routinely acquired clinical data.^20^^,^^21^

To overcome these limitations, we follow a physics-informed learning paradigm: simulate artifacts through medical physics and learn the inverse through neural networks.^16^ Rather than relying on real paired artifact-free data, we reframe distortion correction as learning the inverse of a forward ssEPI distortion process. A forward physical simulator generates corrupted images, and the network learns to restore anatomy from these physics-driven artifacts.

## Materials and Methods

### Dataset

This study evaluated 2 cohorts using 3T prostate MRI: the fastMRI prostate dataset (March 2020 to April 2021), which contained 312 MR examinations, and an in-house Cedars-Sinai cohort (September 2017 to April 2022) comprising 130 prostate MRI examinations (Table 1 and Table S1). To drive the realistic forward ssEPI simulator, we additionally collected 11 B_0_ field maps from patients with unilateral or bilateral hip prostheses. To account for the substantial diversity of clinically encountered pelvic susceptibility fields, these maps served as realistic base templates for a targeted parametric augmentation strategy that generated 110 field maps for metal-induced B_0_ off-resonance patterns in the prostate (see Supplementary Material S2 for details). Among the 442 retrospective MR examinations, a clinical test set of 34 cases (5 fastMRI and 29 Cedars-Sinai) was exclusively curated based on predefined inclusion criteria for geometric distortion (see Supplementary Material for detailed inclusion criteria S3). The remaining 408 MR examinations with no visible susceptibility artifacts (307 fastMRI and 101 Cedars-Sinai) formed the synthetic training pool for generating paired distorted/undistorted data. For this pool, low-b DWI, high-b DWI, ADC maps, and co-registered T_2_W images were used as undistorted references. To ensure statistical comparability with the clinical evaluation, we intentionally matched the size of the clinical test cohort by holding out exactly 34 subjects as the synthetic test set for quantitative evaluation. The remaining 374 subjects were allocated exclusively to the training set for the restoration network.

**Table 1:** Summary of prostate mpMRI datasets and clinical evaluation cohort used in this study.

Cohort	No. examinations	Age summary	Description
FastMRI prostate dataset	312	Mean 65.05 years; median 65; range 39-86	Public source cohort; 5 severe-distortion cases contributed to the clinical evaluation cohort
In-house prostate dataset	130	Mean 66.89 years; median 67; range 47-81	Retrospective institutional cohort; 29 severe-distortion cases contributed to the clinical evaluation cohort
Clinical evaluation (pathology-reference subgroup)	34 (18)	N/A	Severe-distortion cohort used for blinded reader study; 18 cases had pathology-related information

Two cohorts were included: the fastMRI prostate dataset (*n* = 312) and an in-house Cedars-Sinai cohort (*n* = 130), yielding a total of 442 examinations. Age is summarized as mean, median, and range for each cohort. A subset of 34 cases with severe distortion was selected for blinded clinical reader evaluation, of which 18 cases had pathology-related information. mpMRI denotes multiparametric MRI, including T2-weighted (T2W) imaging and diffusion-weighted imaging (DWI). The fastMRI prostate dataset was acquired on Siemens 3T MAGNETOM Vida scanners, and the in-house Cedars-Sinai cohort was acquired on a Siemens 3T Biograph mMR scanner; detailed scanner information is provided in Table S1.

### Preprocessing and forward distortion simulation

All mpMRI sequences from each study were regridded using DICOM geometric information and resampled to a common 256 × 256 in-plane matrix with 0.56 × 0.56 mm^2^ pixel spacing while preserving the original through-plane resolution. This DICOM geometry-based regridding was used to place T2W and diffusion images on a common spatial grid for model input preparation. No image-based rigid or deformable registration was performed; therefore, inter-sequence motion, physiologic deformation, and nonlinear EPI distortion were not explicitly corrected during preprocessing. For each 2D B_0_ slice, a voxel displacement map (VDM) was computed using the standard ssEPI formulation^10^:


$$\mathrm{VDM}=\;S_{\mathrm{pe}}\cdot \Delta f(r)(N_{\mathrm{pe}}\cdot \frac{\mathrm{PF}}{R}-1)/\mathrm{ESP}$$

where $S_{pe}$ is the PE direction (+1/−1), Δf(r) is the off-resonance at pixel r, $N_{pe}$ is the number of PE lines, PF is the partial-Fourier factor, R is the in-plane acceleration factor, and ESP is the echo spacing. These VDMs were applied to distortion-free DWIs by displacing and splatting voxel intensities along the PE direction according to the prescribed shifts, thereby reproducing stretching, compression, pixel pile-up, and local signal dropout characteristic of ssEPI distortion.

**Figure 1:**
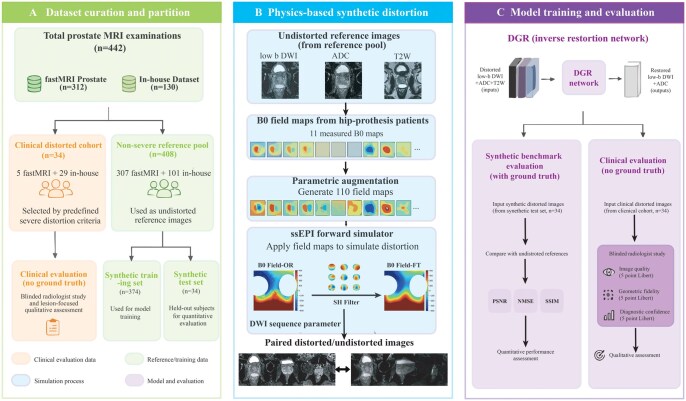
Overview of the distortion-guided restoration (DGR) framework. (A) Dataset curation and partition. A total of 442 prostate MRI examinations (fastMRI: *n* = 312, Siemens 3T MAGNETOM Vida; in-house: *n* = 130, Siemens 3T Biograph mMR) were divided into a clinical distorted cohort (*n* = 34) for reader evaluation and a non-severe reference pool (*n* = 408). The reference pool was further split into training (*n* = 374) and held-out synthetic test sets (*n* = 34). (B) Physics-based synthetic distortion generation. Undistorted low-b DWI, ADC, and T2W images from the reference pool were combined with B_0_ field maps derived from hip-prosthesis patients. After parametric augmentation, the field maps were applied through an ssEPI forward simulator to generate paired distorted/undistorted training data. (C) Model training and evaluation. The DGR model is trained to restore distortion-corrupted diffusion images using T2W anatomical guidance. Performance is evaluated on a synthetic benchmark with ground truth using quantitative metrics (PSNR, NMSE, SSIM), and on a clinical cohort without ground truth using independent blinded assessment of 2 radiologists regarding image quality, geometric fidelity, and diagnostic confidence. Abbreviations: ADC, apparent diffusion coefficient; DWI, diffusion-weighted imaging; NMSE, normalized mean squared error; PSNR, peak signal-to-noise ratio; ssEPI, single-shot echo-planar imaging; SSIM single-shot echo-planar imaging; T2W, T_2_-weighted.

Using this pipeline (Figure 1), we generated more than 40 000 paired distorted/undistorted images from the training-pool subjects by incorporating multiple PE directions (left-right, right-left, anterior-posterior, and posterior-anterior). The synthesized dataset captured a wide spectrum of realistic distortion patterns representative of metal-affected pelvic imaging and served as the primary supervision for training the inverse (restoration) networks.

### Network architecture

**Figure 2:**
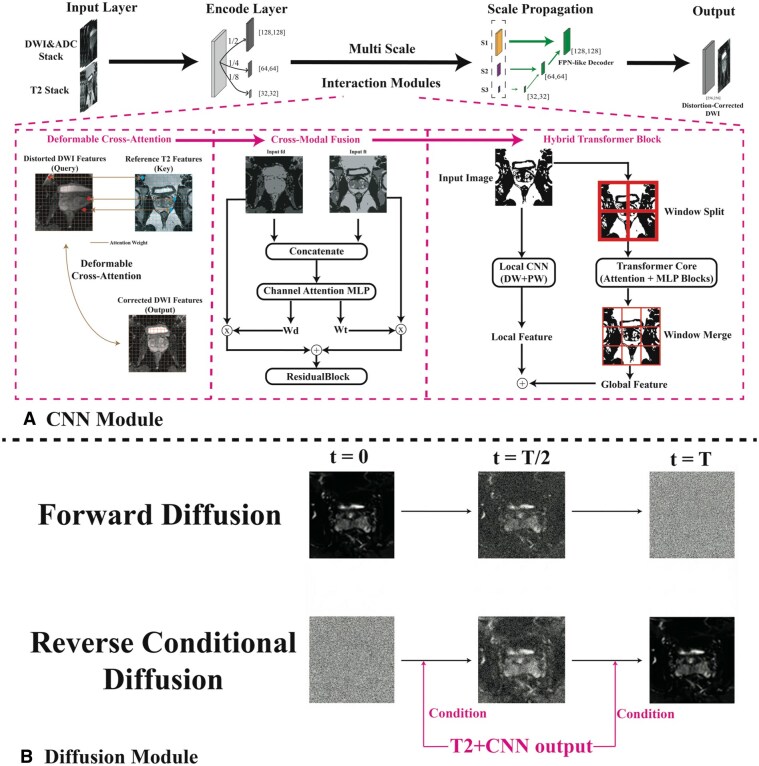
Overview of the proposed inverse restoration network. (A) CNN front-end. A multi-scale encoder with deformable cross-attention, cross-modal fusion, and hybrid transformer blocks extracts geometry-aware features from distorted DWI/ADC inputs under T_2_W anatomical guidance, producing coarse distortion-corrected outputs. (B) Conditional diffusion refinement. During the forward diffusion process, clean images are progressively noised; the reverse conditional process denoises the CNN prediction toward high-fidelity results using T_2_W+CNN features as conditioning signals. This 2-stage design enables physics-guided coarse inversion and diffusion-based texture and detail restoration. Abbreviations: ADC, apparent diffusion coefficient; CNN, convolutional neural network.

Building upon the forward EPI distortion simulator described above, we designed a physics-informed distortion-guided restoration (DGR) network using a hybrid convolutional neural network (CNN)-diffusion architecture to approximate the inverse of the simulated distortion (Figure 2). Given distorted low-b DWI and ADC maps, along with an aligned T_2_W image, the network predicts corrected low-b DWI and ADC maps and calculates the high-b DWI from them. We chose low-b DWI and ADC, rather than high-b DWI, as restoration targets because high-b images have substantially lower SNR and are more strongly affected by noise, making them less suitable for our physics-based distortion synthesis and supervised restoration framework.

The CNN front-end operates as a coarse, physics-aligned inverse model. Stacks of distorted low-b DWIs and ADC are processed jointly with the registered T_2_W image by a compact multi-scale encoder-decoder.^22^ The CNN aggregates geometry-aware features across scales and uses T_2_W-conditioned attention to encourage anatomical consistency between the corrected diffusion contrast and the reference anatomy.^23^^,^^24^ This branch produces initial low-b and ADC predictions that already remove most bulk distortion but remain overly smooth in severely corrupted regions, particularly on ADC where realistic noise patterns are hard to reproduce. To restore fine texture and more natural signal statistics, we added a conditional diffusion refinement module that operates on the CNN outputs rather than generating images from pure noise.^25^ During training, a diffusion UNet receives a noisy version of the undistorted low-b/ADC pair together with the frozen CNN predictions and the co-registered T_2_W image as conditioning. At inference, the process is initialized from the CNN output and perturbed with a small amount of noise (SDEdit-style), allowing the diffusion model to sharpen edges and reinstate realistic noise behavior while preserving the overall anatomy provided by the CNN and T_2_W.^26^ The CNN is supervised with a weighted L1+SSIM loss, whereas the diffusion stage follows a standard DDPM-style clean-image prediction objective. Detailed architectural configurations, training schedules, and ablation studies are provided in the Supplementary Material S4.

### Evaluation

The proposed method DGR was evaluated using both synthetic and clinically acquired datasets.

#### Synthetic datasets (34 subjects)

Quantitative evaluation used peak SNR (PSNR), structural similarity index (SSIM), and normalized mean squared error (NMSE) computed between corrected images and their undistorted ground-truth (GT) references. In addition, for evaluation only, a reverse phase-encoded distorted counterpart was generated to enable conventional B_0_-guided methods (eg, FSL, TOPUP) to run under their required dual-encoding input.

For comparison with classical correction pipelines, both FSL FUGUE and TOPUP were configured under idealized conditions. For FUGUE, the GT field map produced by the forward simulator was supplied directly, bypassing the noise, phase-unwrapping artifacts, and sequence mismatch that typically degrade field-map-based correction in vivo. Thus, the reported FUGUE performance represents an upper bound rather than typical clinical performance. Similarly, TOPUP was provided with the GT distortion field in place of its own estimated field map, isolating its reconstruction behavior from field-estimation uncertainty and representing the theoretical limit of reverse PE correction. The default TOPUP configuration, in which the field map is estimated from the forward/reverse ssEPI pair, was also tested.

#### Clinical datasets (34 subjects)

Qualitative evaluation was performed on the clinical test cohort, where undistorted DWI is not obtainable in vivo. Therefore, co-registered T_2_W images were used as a soft anatomical reference. The model received the distorted low-b DWI, ADC map, and T_2_W as inputs. Two abdominal radiologists (each with more than 5 years of experience) independently scored each dataset on 3 separate 5-point Likert scales (1 = poor, 5 = excellent) for geometric fidelity, overall image quality, and diagnostic confidence, using the detailed criteria defined in the Supplementary Material S5. Among the 34 subjects, 18 had a history of prostate abnormality, and 16 had imaging-identified, histologically confirmed lesions; this clinical information was used to contextualize lesion-level findings in the “Results and Discussion”.

Statistical analysis was performed using non-parametric paired comparisons (Wilcoxon signed-rank test) to account for the ordinal nature of the Likert scale. A *P*-value < .05 was considered statistically significant.

#### Code availability

The code for this study will be made publicly available at https://github.com/Albertlongzi/DGR upon publication.

## Results

### Synthetic experiments with undistorted GT

Quantitative results are summarized in Table 2. Across both low-b (*b* = 50 s/mm^2^) DWI and ADC maps, the proposed DGR model achieved the highest PSNR and the lowest NMSE among all methods, while maintaining competitive SSIM. Specifically, DGR reached 23.88 ± 2.93 dB/0.089 ± 0.049 (PSNR/NMSE) for low-b DWI and 22.99 ± 1.97 dB/0.062 ± 0.028 for ADC, outperforming both FUGUE and TOPUP approaches. Although FUGUE yields the highest SSIM under ideal conditions, DGR maintains comparable SSIM values while substantially reducing residual reconstruction error.

**Table 2:** Quantitative comparison of distortion-correction methods on the synthetic dataset.

Method	Contrast	PSNR (dB)	NMSE	SSIM
Baseline	*b* = 50 ADC	17.67 ± 4.06 15.60 ± 2.51	0.447 ± 0.355 0.351 ± 0.172	0.564 ± 0.146 0.281 ± 0.115
FUGUE + Fieldmap^a^	*b* = 50 ADC	17.92 ± 7.11 22.37 ± 2.51	0.903 ± 1.334 0.073 ± 0.040	**0.770 ± 0.094 0.736 ± 0.065**
TOPUP + Rev PE^a^	*b* = 50 ADC	22.45 ± 4.83 19.42 ± 2.92	0.175 ± 0.181 0.157 ± 0.107	0.705 ± 0.115 0.409 ± 0.140
DGR	*b* = 50 ADC	**23.88 ± 2.93 22.99 ± 1.97**	**0.089 ± 0.049 0.062 ± 0.028**	0.706 ± 0.079 0.624 ± 0.107

PSNR (peak signal-to-noise ratio), NMSE (normalized mean squared error), and SSIM (structural similarity index) were computed between the restored images and the undistorted reference for both low-b (*b* = 50 s/mm^2^) DWIs and ADC maps. Values are reported as mean ± SD across the synthetic test set (*n* = 34). Higher PSNR and SSIM values and lower NMSE values indicate better agreement with the reference. Distortion-guided restoration achieved the highest PSNR and lowest NMSE across both contrasts. Bold values indicate the best-performing result for each metric and contrast.

a FUGUE and TOPUP are evaluated under an ideal setting that requires additional acquisitions (dual-echo GRE field maps or reverse phase-encoding DWI pairs) and high-quality distortion fields. Distortion-guided restoration operates on the routine mpMRI protocol without extra acquisitions.

Abbreviations: ADC, apparent diffusion coefficient; DGR, distortion-guided restoration; FUGUE, FMRIB’s Utility for Geometrically Unwarping EPI; NMSE, normalized mean squared error; PE, phase-encoding; PSNR, peak signal-to-noise ratio; SSIM, structural similarity index; TOPUP, Tool for Estimating and Correcting Susceptibility-Induced Distortions.

These improvements in PSNR and NMSE were statistically significant when compared with TOPUP and/or FUGUE across both contrasts (paired Wilcoxon signed-rank tests, all *P* < .001 except low-b PSNR vs TOPUP, *P* = .02), whereas no significant difference in SSIM was observed between DGR and TOPUP for low-b DWI (*P* = .95).

For reference, when TOPUP relied on its own estimated field map rather than the ideal situation, SSIM dropped to 0.572 ± 0.200 and 0.374 ± 0.202 for low-b DWI and ADC, respectively, and NMSE increased to 0.227 ± 0.196 and 0.193 ± 0.121, underscoring the difficulty of robust field estimation in highly distorted pelvic imaging.

**Figure 3:**
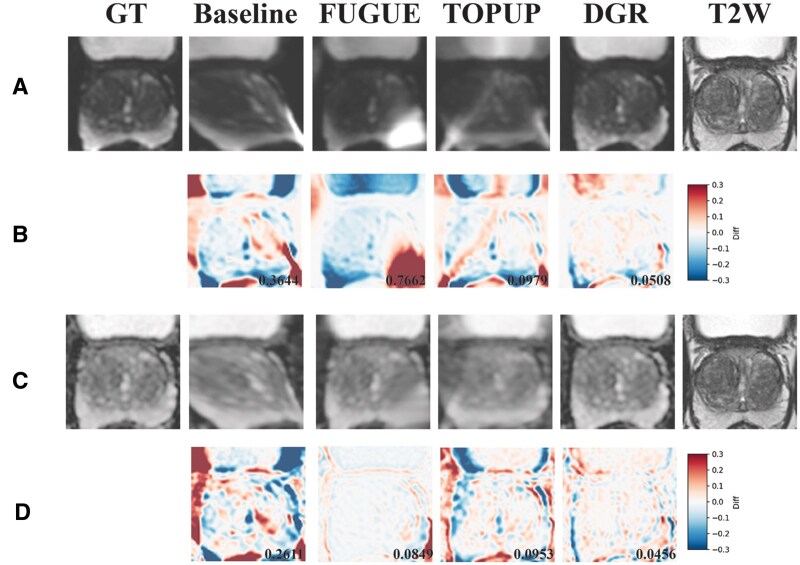
Representative synthetic example comparing distortion-correction methods. (A) Corrected low-b (*b* = 50 s/mm^2^) DWIs produced by each method, with the undistorted ground truth (GT) and co-registered T_2_W shown for reference. (B) Corresponding voxel-wise difference maps (DWI-GT), with normalized mean squared error (NMSE) labeled. (C) Corrected ADC maps and their difference maps (bottom row). (D) Corresponding voxel-wise difference maps for ADC (ADC-GT), with NMSE labeled. The proposed DGR method achieves substantially lower reconstruction error and improved geometric fidelity compared with the baseline, FUGUE, and TOPUP. However, subtle signal variation in low-texture regions such as the bladder may be smoothed in the DGR output, indicating that improved geometric restoration does not necessarily imply perfect preservation of all local signal variations. Abbreviations: DGR, distortion-guided restoration; FUGUE, FMRIB’s Utility for Geometrically Unwarping EPI; GT, ground truth; TOPUP, Tool for Estimating and Correcting Susceptibility-Induced Distortions; T2W, T_2_-weighted.

A representative synthetic example is shown in Figure 3. Visually, DGR demonstrates closer geometric alignment to the undistorted GT, compared with the baseline, FUGUE, and TOPUP. The error maps further quantify these gains: for *b* = 50 DWI, DGR achieves the lowest voxel-wise error (0.0508 vs.∼0.3644, 0.7662, and 0.0979), and similarly for ADC (0.0436 vs.∼0.2761, 0.0849, and 0.0935).

### Clinical experiments

**Figure 4:**
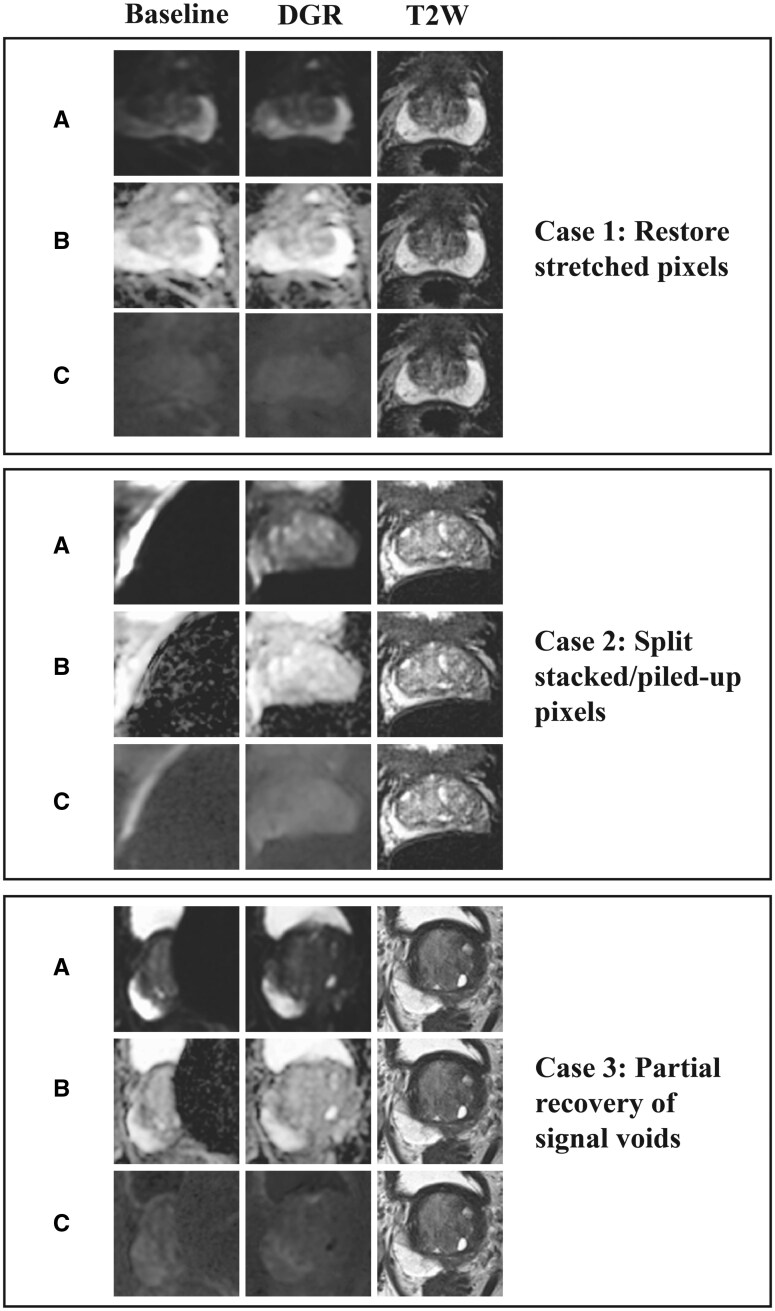
Clinical examples demonstrating the generalization ability of the proposed DGR model across diverse distortion patterns. Each block shows (A) low-b DWI (*b* = 50 s/mm^2^), (B) ADC maps, and (C) high-b DWI (*b* = 1400 s/mm^2^) for the baseline ssEPI reconstruction, the proposed DGR output, and the corresponding co-registered T_2_W reference. Case 1 illustrates recovery of severely stretched pixels; case 2 shows splitting of stacked/piled-up regions into anatomically plausible structures; case 3 demonstrates partial recovery of signal and improved boundary definition in regions affected by extreme local signal voids. Abbreviations: DGR, distortion-guided restoration; T2W, T_2_-weighted.

Figure 4 shows representative real clinical cases illustrating that DGR generalizes across multiple common ssEPI failure modes, including (i) large-scale geometric stretching, (ii) localized pile-up/splitting of compressed structures, and (iii) partial recovery in regions affected by severe signal voids.

**Figure 5:**
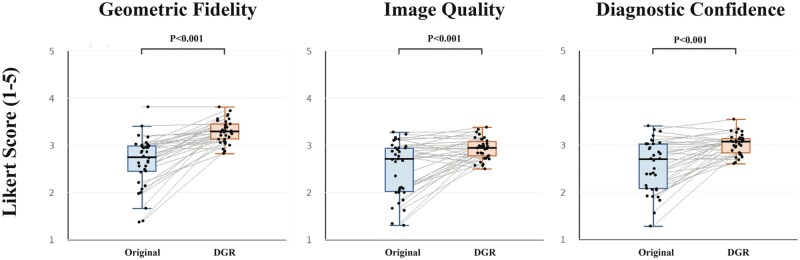
Scores of 2 independent radiologists for original versus DGR-processed prostate diffusion images. Boxplots show 5-point Likert ratings (1 = poor, 5 = excellent) for (left) geometric fidelity, (middle) image quality, and (right) diagnostic confidence in 34 clinical ssEPI MR examinations. For each subject, readers scored the original diffusion images and the corresponding DGR-corrected images. Black dots represent per-subject averages across all evaluated slices, and gray lines connect paired scores from the same subject to visualize individual improvements. *P*-values from paired Wilcoxon signed-rank test demonstrate significant improvements with DGR for all 3 metrics. Abbreviation: DGR, distortion-guided restoration.

At the group level, DGR improved reader-perceived image quality and diagnostic usability across the 34 clinical subjects with susceptibility-induced distortion (Figure 5). Two blinded radiologists assigned systematically higher scores to DGR-processed images than to the original ssEPI, with median geometric fidelity increasing from 2.74 ([IQR]: 2.43-2.98) to 3.29 (3.12-3.44), overall image quality from 2.70 (2.01-2.93) to 2.94 (2.76-3.07), and diagnostic confidence from 2.69 (2.07-3.02) to 3.06 (2.82-3.14) on a 5-point Likert scale (all *P* < .001; Table 3). To account for the ordinal nature of the Likert scale, interrater agreement metrics are provided in Supplementary Material S6.

**Table 3:** Radiologist qualitative assessment of original ssEPI (single-shot echo-planar imaging) and DGR (distortion-guided restoration)-processed images.

Metric	Original ssEPI	DGR
Geometric fidelity	**2.74** (2.43-2.98)	**3.29** (3.12-3.44)
Image quality	**2.70** (2.01-2.93)	**2.94** (2.76-3.07)
Diagnostic confidence	**2.69** (2.07-3.02)	**3.06** (2.82-3.14)

Scores for geometric fidelity, overall image quality, and diagnostic confidence are reported as median (IQR) on a 5-point Likert scale (1 = poor, 5 = excellent), based on independent blinded evaluation of 34 clinical cases by 2 radiologists. Higher scores indicate improved perceived image quality and diagnostic usability. Abbreviations: DGR, distortion-guided restoration; ssEPI, single-shot echo-planar imaging. Bold values indicate the median scores, with interquartile ranges (IQRs) shown in parentheses.

**Figure 6:**
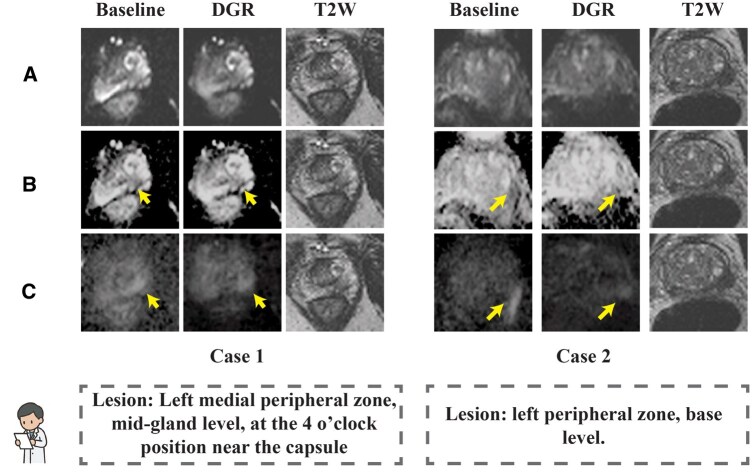
Lesion-related evaluation across 2 representative clinical cases with severe susceptibility distortion. For each case, columns show the baseline ssEPI, the proposed DGR correction, and the co-registered T_2_W reference. (A) Low-b DWI (*b* = 50 s/mm^2^), (B) ADC map, and (c) high-b DWI (*b* = 1400 s/mm^2^). Arrows indicate the tumor location on ADC and high-b DWI images. Case 1: artifact-dominated distortion with severe geometric stretching. Case 2: Lesion displacement due to extreme susceptibility-induced warping. Abbreviations: DGR, distortion-guided restoration; T2W, T_2_-weighted.

Beyond global image quality, we further examined lesion-related visualization in selected clinical cases (Figure 6). Qualitatively, DGR consistently preserved intrinsic diffusion contrast while mitigating metal-related blooming and geometric stretching (case 1). In scenarios with severe susceptibility artifacts where lesions were artifactually shifted outside the prostate contour, DGR successfully restored anatomical alignment, matching the co-registered T2W images and biopsy findings (case 2).

In the subset of subjects with available histopathologic reference (*n* = 18), DGR introduced zero diagnostic errors. Specifically, DGR yielded no false negatives (ie, suppression of confirmed lesions) and generated no new false-positive findings. Among the 16 subjects (16/16, 100%) with histologically confirmed lesions, DGR preserved lesion conspicuity in all instances and increased the diagnostic confidence score by > 0.5 in 4 (4/16, 25%) of these cases.

## Discussion

In this study, we introduced a physics-informed hybrid CNN-diffusion framework DGR that leverages realistic forward physical simulation and T2W anatomical conditioning to reduce susceptibility-induced ss-EPI distortions. Across synthetic benchmarks and a curated clinical cohort, this approach demonstrated improved geometric alignment and lesion interpretability without requiring an additional field map acquisition. Our findings demonstrated the feasibility of a learning approach that enables data-driven inversion of a realistic forward-distortion model, serving as a promising alternative to conventional distortion correction pipelines, particularly when mitigating extensive susceptibility artifacts where traditional analytical methods break down.

At a mechanistic level, conventional correction approaches struggle with severe geometric deformation because they rely on analytical inversions that become mathematically ill-posed. As illustrated in our synthetic experiments (eg, Figure 3), these traditional pipelines exhibit distinct failure modes. For instance, field-map-based methods like FUGUE often introduce unnatural hyperintense patches on low-b DWI and excessive smoothing on ADC maps. This is a direct consequence of inaccurate pixel remapping and aggressive Jacobian intensity modulation when attempting to compensate for severe signal pile-up. Conversely, although TOPUP avoids such extreme localized artifacts, it often produces globally blurred, veil-like reconstructions with softened boundaries and diminished granular structural detail.

In contrast, DGR avoids explicit calculation of a deformation field. Instead, it learns a data-driven restoration that jointly leverages diffusion images (low-b DWI and ADC) and a paired T_2_W image as an anatomical constraint, which is particularly valuable in regions where pile-up or signal voids render analytical inversion ill-posed. To ensure T2W conditioning remains robust across varying clinical protocols, we explicitly mixed multiple contrast variants (eg, fat-suppressed and non-fat-suppressed) during training and introduced a contrast-aware latent representation within the CNN module, stabilizing the network’s response to diverse contrast shifts.

From a practical perspective, the elimination of the requirement for a B_0_ field map represents a key advantage of the proposed framework. By avoiding additional acquisitions, DGR reduces scan time and mitigates failure modes associated with motion, misregistration, or poor field-map quality (factors that are particularly relevant in prostate imaging with metal implants or rectum distention).

Several limitations warrant consideration. First, the current clinical reader study primarily supports improved perceived image quality, rather than proven routine clinical utility. The study did not evaluate its effect on clinical metrics, such as lesion detection, biopsy targeting yield, interpretation time, or impact on treatment decision-making. Future studies are needed to determine whether improved image quality translates into measurable clinical benefit. Second, the evaluation was conducted on a cohort of restricted size and a limited number of scanning platforms, particularly for pathology-referenced lesion-level assessment. Although the full clinical evaluation included 34 cases with severe susceptibility-related distortion, only 18 cases had available pathology-related clinical information, and 16 had imaging-identified, histologically confirmed lesions. Larger prospective, multi-center studies with systematic histopathology reference standards are needed to estimate reliable rates of false positives, false negatives, diagnostic failure, and hallucination rates, and to determine whether the observed improvements are clinically meaningful. Third, our forward simulator was driven primarily by templates from hip-implant patients. Despite applying physics-informed perturbations, the training distribution may under-represent complex susceptibility patterns dominated by dynamic rectal distension. Expanding the template library to encompass these variations remains a priority for future work. Finally, our simulation and restoration were performed in a 2D slice-wise setting, which does not fully capture through-plane distortions or enforce strict 3D consistency across adjacent slices. Because preprocessing used DICOM geometry-based regridding rather than image-based registration, residual T2W-DWI/ADC mismatch from inter-sequence motion, physiologic changes, or through-plane differences may remain. Such non-distortion-related mismatch may weaken T2W anatomical guidance and remains a limitation of the current approach.

Because DGR incorporates a generative diffusion module, hallucination risk remains an important concern despite the conservative img2img (0.1) strength used to minimize structural deviation. In regions with severe signal dropout, the original DWI may not contain sufficient information to recover the true underlying signal, making restoration more dependent on learned priors and T2W anatomical conditioning. Such cases should be considered outside the reliable interpretive scope of the present method; a representative signal-limited example, excluded from the main lesion-related evaluation, is shown in Figure S1. These findings motivate future work on uncertainty estimation, failure detection, and 3D contextual modeling. Although repeated inference showed minimal stochastic variability, reproducible outputs do not rule out the potential of systematic hallucination or local over-smoothing; therefore, severely signal-limited DGR-processed regions should be interpreted with caution.

## Conclusion

We introduced a physics-informed learning framework that restores prostate DWI distorted by ssEPI through a hybrid CNN-diffusion architecture trained on large-scale, simulated forward models. Across a small proof-of-concept cohort of synthetic benchmarks and real clinical cases, the method can improve distortion recovery and diagnostic interpretability, outperforming conventional methods for image quality without requiring additional acquisitions such as B_0_ field maps or reverse PE scans. By learning a robust inverse mapping grounded in physical distortion mechanisms, the proposed DGR framework, if further validated in larger cohorts and in a clinical context, offers a practical and scalable solution that could be developed to improve prostate DWI quality in a clinical setting, particularly in metal-affected imaging where conventional methods often fail.

## Supplementary Material

umag031_Supplementary_Data

## Data Availability

The data supporting the findings of this study are available from the corresponding author upon reasonable request. The code will be available on GitHub upon acceptance.

## Abbreviations

ADC: apparent diffusion coefficient
CNN: convolutional neural network
DGR: distortion-guided restoration
DWI: diffusion-weighted imaging
IQR: interquartile range
NMSE: normalized mean squared error
PSNR: peak signal-to-noise ratio
ssEPI: single-shot echo-planar imaging
SSIM: structural similarity index
VDM: voxel displacement map
