# Supplementary material for: Let distortion-guided restoration: a physics-informed learning framework to correct prostate diffusion MRI artifacts
Source: Radiol Adv. 2026 Jul 14;3(4):umag031. doi: 10.1093/radadv/umag031 (PMC13446218; doi:10.1093/radadv/umag031)
Supplement: Supplementary file 1 [file umag031_supplementary_data.zip › Supplementary_Data.zip!/The_supplementary_material_for_DGR.pdf]

The supplementary material for DGR

S1. Detailed MRI Acquisition Protocols

	Scanner		$TR_{(s)}$	$TE_{(ms)}$	$FOV_{(mm)}$	Matrix Size	Slice $Thickness_{(mm)}$
**FastMRI Prostate Dataset**	Siemens's Vida	*T2W*	3.5-7.2	100	180 × 180	320 × 320	3
		DWI	5.0-7.3	77	200 × 200	100 × 100	3
**In-House Prostate Dataset**	Siemens's mMR	*T2W*	4.0-5.5	80-132	160 × 160	256 × 256	3
		DWI	4.5-5.0	73-95	200 × 200	256 × 256	3

Table S1: **Core acquisition parameters for the fastMRI and in-house prostate MRI datasets.** Scanner type and sequence parameters are reported for T2-weighted imaging (T2W) and diffusion-weighted imaging acquired using single-shot echo-planar imaging (DWI, ssEPI). TR = repetition time; TE = echo time; FOV = field of view.

Additional Sequence Parameters

Beyond the parameters listed in Table S.1, all T_2-weighted (T2W) images were acquired as 2D sequences (e.g., 2D Turbo Spin Echo), and all DWIs were acquired using 2D single-shot echo-planar imaging (ss-EPI).

Diffusion Acquisition Harmonization Across Datasets

The two prostate MRI cohorts shared the same low-b diffusion acquisition (b = 50 s/mm²), but differed in the available high-b images: the fastMRI Prostate dataset used b = 1000 s/mm², whereas the in-house Cedars–Sinai cohort used b = 1400 s/mm². In the proposed restoration framework, low-b DWI and ADC maps were used as the primary restoration targets together with the co-registered T2W image. High-b DWI was not used as a direct restoration target; instead, it was synthesized from the restored low-b DWI and ADC according to the diffusion signal relationship. Additionally, we used the scanner-provided ADC maps available for each cohort, rather than recomputing them from raw diffusion signals.

Field Map Acquisition

We utilized a 2D 4-echo gradient-recalled echo (GRE) sequence (3.1mm×3.1mm×3.1mm) to capture the susceptibility-induced field variations. The multiple echo times (TEs) were uniformly spaced at 2.3, 4.6, 6.9, and 9.2ms. The acquired multi-echo phase data were subsequently processed using an in-house phase unwrapping script developed in MATLAB (MathWorks, Natick, MA) to calculate the B_0 field map in Hertz.

S2. Parametric B0 field map augmentation

To mitigate overfitting and effectively expand the diversity of local susceptibility artifacts for training, we applied a targeted parametric mathematical augmentation strategy to the 11 acquired in vivo field maps.

Specifically, each base map was parameterized using a 12th-order spherical harmonic expansion. To generate diverse field variants while preserving the realistic global field geometry of the patients, we kept the low-order coefficients (orders 0 through 2) unaltered. These low-order terms represent the bulk magnetic field structure, such as global offsets and linear gradients. We then perturbed higher spatial-frequency components, which can represent more localized and complex susceptibility variations such as those associated with bowel gas or implant-related field perturbations.

For these higher-order terms, Gaussian noise was added relative to the median magnitude of the coefficients within each order. We utilized 10 adjustment factors (ranging from 0 to 0.15) to scale the perturbation strength. This process generated 10 unique field variants per base subject, resulting in an augmented pool of 110 field maps. Empirically, this localized high-order perturbation produced a continuous spectrum of field inhomogeneities, with the root-mean-square (RMS) deviation from the baseline field scaling monotonically from approximately 30 Hz (at factor 0.03) to 150 Hz (at factor 0.15). Thus, the augmented maps preserved the dominant spatial structure of measured susceptibility fields while introducing controlled local variation, rather than representing random distortion patterns.

S3. Inclusion and exclusion criteria for the clinical test set

To rigorously evaluate the proposed method on clinically challenging cases, the 34 subjects in the clinical test set were prospectively selected from the available 444 studies based on their baseline geometric distortion severity by experienced reader. The inclusion and exclusion criteria were defined as follows:

Inclusion Criteria

1. Availability of paired T2W and ss-EPI DWI sequences.
2. Presence of marked to severe geometric distortion on baseline DWI. This was quantitatively defined as a score of 1 or 2 on our 5-point geometric fidelity Likert scale (1 = Severe distortion; 2 = Marked distortion). The full definitions of the 5-point scale are detailed in the Reader Study section.

Exclusion Criteria

1. Cases exhibiting moderate to minimal geometric distortion (Likert scores 3, 4, or 5).

S4. Network training details

CNN restoration backbone.

The convolutional front-end of the DGR framework was trained on the synthetic paired dataset using 2.5D input stacks (radius = 2 slices). For each sample, the inputs consisted of distorted low-b DWI and ADC maps concatenated with the co-registered T2W image. Each channel was first processed by a shallow full-resolution stem, followed by a compact multi-scale encoder with residual blocks and stride-2 down-sampling, yielding a feature pyramid from fine to coarse resolutions.

At each scale, a contrast-aware deformable cross-attention module treated the distorted DWI features as queries and the T2W features as keys/values, predicting adaptive spatial offsets and attention weights to sample anatomically corresponding T2W features despite geometric mismatch caused by distortion. The attended features were refined by lightweight transformer-style blocks that combined local self-attention with convolutional feed-forward layers, and a

channel-wise fusion module re-weighted DWI and T2W channels to balance anatomical priors with diffusion contrast.

An FPN-style top-down path aggregated multi-scale features back to full resolution and produced initial CNN predictions for low-b DWI and ADC. Auxiliary outputs at reduced resolutions were supervised to stabilize training, and their losses were added to the full-resolution loss with decreasing weights.

The CNN restoration backbone was trained jointly on low-b DWI and ADC targets, using 2.5D stacks with a batch size of 6 for 15 epochs. The optimizer used an initial learning rate of 3×10^{-4} with linear warm-up for 3,054 steps followed by a square-root learning-rate decay schedule. Gradients were clipped at an ℓ_2-norm of 1.0.

The training objective combined an ℓ_1 reconstruction term with a multiscale SSIM regularizer,

$$\mathcal{L}_{cnn} = |\hat{x} - x|_1 + \lambda_{\mathrm{SSIM}}\left(1 - \mathrm{SSIM}(\hat{x}, x)\right),$$

with λ_{SSIM} =0.25. Auxiliary predictions from intermediate decoder scales were supervised with the same loss and added with weights 0.2 and 0.05, respectively. Losses were averaged over the low-b DWI and ADC contrasts.

Diffusion refinement module.

The diffusion refinement module was trained separately after freezing the CNN backbone. Its goal was to correct residual artifacts and restore fine-scale texture rather than reconstruct images from scratch.

A conditioning branch encoded the T2W image together with the frozen CNN predictions (low-b and ADC) using shallow convolutional stems, deformable cross-attention, and a hybrid transformer block, producing a compact T2W+CNN conditioning feature map. During training, this conditioning map was concatenated with a noisy version of the undistorted low-b/ADC pair and fed into an SR3-style UNet diffusion backbone trained under a DDPM framework with "clean-image" prediction (prediction_type = "sample"). The diffusion objective was

$$\mathcal{L}_{\text{diff}} = E_{t,\epsilon} \left\| \widehat{x_0^{(t)}} - x_0 \right\|_2^2,$$

where $\widehat{x_0^{(t)}}$ is the network's estimate of the clean image at diffusion step t and x_0 is the ground-truth undistorted image.

Training used a batch size of 6 for 100 epochs with a learning rate of 1×10^{-4}, 1,000 warm-up steps, gradient clipping at norm 1.0, and no gradient accumulation. We adopted a standard DDPM noise schedule and trained with random diffusion steps sampled uniformly over the trajectory. Validation was performed using a fixed img2img strength of 0.3 and a DPM-Solver sampler.

At inference time, following the SDEdit formulation, the diffusion process was initialized with the CNN prediction instead of pure noise. The CNN output was perturbed with a moderate noise level (img2img strength = 0.1) before running the reverse diffusion process. This procedure preserved the coarse structure and geometry recovered by the CNN while allowing the diffusion model to sharpen edges, reintroduce realistic noise and texture—particularly in ADC—and resolve residual artifacts such as pile-up and local signal voids under T2W-guided conditioning.

Ablation study

To better understand the contribution of the learnable components in DGR, we performed an ablation study on the synthetic paired test set. The purpose of this analysis was to separate the effect of the CNN restoration backbone from that of the diffusion refinement stage.

The physics-based forward simulator was not included in the ablation because it is not a test-time restoration block. Instead, it serves as the data-generation mechanism used to synthesize paired distorted/undistorted training examples. Therefore, the ablation focused on the restoration network itself.

We compared two settings: (1) CNN only, in which the multi-scale T2W-guided CNN produced the final restored output directly; and (2) CNN + Diffusion, corresponding to the full DGR model, in which the CNN prediction was further refined by the conditional diffusion stage.

Quantitative results are summarized in Table S2. For low-b DWI (b = 50 s/mm²), adding diffusion refinement improved all evaluated metrics, including PSNR, NMSE, SSIM, LPIPS, and Tenengrad ratio. For ADC, the CNN-only backbone remained slightly better on PSNR/NMSE/SSIM, whereas the full model achieved clearly better LPIPS and Tenengrad ratio. These findings suggest that the diffusion stage mainly contributes improved perceptual fidelity and sharper texture/detail recovery, while preserving competitive quantitative restoration performance.

We also explored a no-T2W-guidance setting by replacing the T2W input with zeros during training. Under this condition, optimization became unstable, and the model failed to converge to a meaningful restoration solution. Because this setting did not yield a valid trained model, we do not report it as a formal quantitative ablation. Nevertheless, this failure mode suggests that T2W guidance is important for stable restoration learning in the current framework, particularly when the network must recover anatomy from severely distorted diffusion inputs.

Method	Contrast	PSNR(dB)	NMSE	SSIM	LPIPS	TENEGRAD RATIO
CNN only	b=50	22.97±2.75	0.108±0.057	0.676±0.078	0.230±0.063	0.734±0.101
	ADC	**23.19±1.89**	**0.059±0.026**	**0.627±0.101**	0.267±0.091	0.746±0.091
CNN + Diffusion	b=50	**23.88±2.93**	**0.089±0.049**	**0.706±0.079**	**0.126±0.035**	**0.847±0.080**
	ADC	22.99±1.97	0.062±0.028	0.624±0.107	**0.151±0.040**	**0.865±0.038**

***The best results**

Table S2. **Ablation study of the learnable restoration network on the synthetic paired test set.** Lower NMSE and LPIPS indicate better performance, whereas higher PSNR, SSIM, and Tenengrad ratio indicate better performance. The Tenengrad ratio quantifies sharpness consistency with the reference image, and LPIPS quantifies perceptual similarity.

S5. Reader Study and Scoring Criteria

Two radiologists independently reviewed the images. For each case, the original ssEPI and DGR-processed images were presented as paired sets (A/B), with randomized order across cases.

Geometric fidelity

Prompt to readers: "Please evaluate the degree of geometric fidelity in the image (including stretching, compression, pile-up, and signal loss), and whether the overall anatomic positioning appears reasonable."

5-point Likert scale:

1 – Severe distortion: Marked misalignment, stretching, compression, or pile-up of anatomy; some regions are almost impossible to interpret (non-diagnostic).

2 – Marked distortion: Obvious distortion that interferes with diagnosis, although the main structures can still be roughly identified.

3 – Moderate distortion: Distortion is present, but images remain diagnosable; several regions show noticeable local deformation.

4 – Mild distortion: Most structures are in a reasonable anatomic position, with only minor local deformation.

5 – Minimal / no distortion: Geometry is close to normal anatomy with no, or only extremely subtle, deformities.

Overall image quality

Prompt to readers: "Please rate the overall image quality, taking into account noise level, artifacts, and overall readability."

5-point Likert scale:

1 – Very poor: Extreme noise and/or aliasing.

2 – Poor: Clearly suboptimal quality.

3 – Acceptable: Image quality is adequate.

4 – Good: Little noise or artifact.

5 – Excellent: High image quality with clear detail, sufficient contrast, and no obvious artifacts.

Diagnostic confidence

Prompt to readers: "How confident are you in your overall diagnostic assessment of the prostate and surrounding structures (including the presence or absence of suspicious lesions) based on this image?"

5-point Likert scale:

1 – Almost no confidence: A reliable diagnosis is essentially impossible.

2 – Low confidence: Diagnosis is very uncertain; only very coarse judgments can be made.

3 – Moderate confidence: A basic diagnosis can be made, but there is limited confidence in several regions.

4 – High confidence: Overall diagnostic confidence is high, with only a few areas of doubt.

5 – Very high confidence: Both global anatomy and lesion assessment are clear, with strong overall diagnostic confidence.

S6. Detailed Reader Study Statistics and Interrater Agreement

Interrater Agreement

Interrater agreement between the two blinded radiologists (pooled across both conditions) was evaluated using quadratic-weighted Cohen's kappa (κ) and Spearman's rank correlation (ρ). Agreement was moderate for geometric fidelity ($\kappa = 0.55$, $\rho = 0.58$; exact agreement rate: 47.2%) and diagnostic confidence ($\kappa = 0.48$, $\rho = 0.46$; rate: 41.8%), and fair for overall image quality ($\kappa = 0.21$, $\rho = 0.39$; rate: 19.4%).

Note on absolute agreement: The fair absolute agreement in overall image quality primarily reflects typical inter-reader bias, wherein readers anchor their subjective baseline scores differently. Crucially, despite differing absolute baselines, both readers independently recognized a consistent relative improvement in the DGR-processed images over the uncorrected ssEPI, confirming the robustness of the restoration across different subjective evaluation thresholds.

S7. The "Gold Standard" Paradox in Severe Susceptibility Distortion

A fundamental challenge in validating distortion correction for pelvic MRI is the absence of perfectly paired distortion-free *in vivo* ground truth. While phantom studies or controlled *in vivo* surrogate experiments (e.g., placing a metallic object near a healthy volunteer) are valuable in standard imaging, they are fundamentally insufficient for validating T2W-guided anatomical restoration in the prostate, due to the following reasons:

1. Lack of Realistic Tissue Contrast: Physical phantoms, even those with diffusion-restricted inserts, cannot replicate the complex, multi-compartmental architecture of the human prostate (e.g., the intricate boundaries between the peripheral zone, transition zone, and neurovascular bundles). Because DGR relies on the rich anatomical priors from the T2W images, testing it on a structurally simplistic phantom would fail to evaluate the network's core capability of preventing hallucination and preserving true anatomical texture.

2. Inaccurate Topography: Placing a titanium surrogate externally on a healthy volunteer induces a macroscopic, low order offset. However, it entirely fails to replicate the high-spatial-frequency, localized field gradients caused by internal bowel gas and surgically embedded prostheses interacting directly with surrounding deep pelvic tissues. The physical boundary conditions and the resulting non-linear spatial deformations are vastly different.

Due to this "Gold Standard Paradox," relying on a physics-informed synthetic simulator (despite its inherent domain bias) remains the most rigorous available method to establish a quantitative baseline, which must then be complemented by thorough qualitative and diagnostic evaluation on real clinical cohorts (as performed in our reader study).

S8. Comparison with other deep-learning method

This study deliberately focused comparisons on established clinical pipelines (FUGUE, TOPUP) rather than recent deep learning (DL)-based EPI correction methods. The rationale for this omission is twofold. First, many existing DL approaches (e.g., standard image-to-image UNets

or GANs) rely on relatively distortion-free standard acquisitions as absolute ground truth for supervised training—an assumption that is physically unattainable for severe pelvic susceptibility artifacts. Second, the vast majority of these networks are strictly optimized for brain DWI. The prostate imaging environment is characterized by vastly different tissue contrasts, dynamic surrounding gas interfaces (rectum/bowel), and massive implant-induced field gradients. Directly applying brain-optimized networks to such extreme pelvic deformations would yield an unfair and uninformative comparison. Benchmarking DGR against other state-of-the-art generative frameworks will be more appropriate in future studies when our method is extended and adapted to brain imaging applications.

S9. Challenging signal-limited example

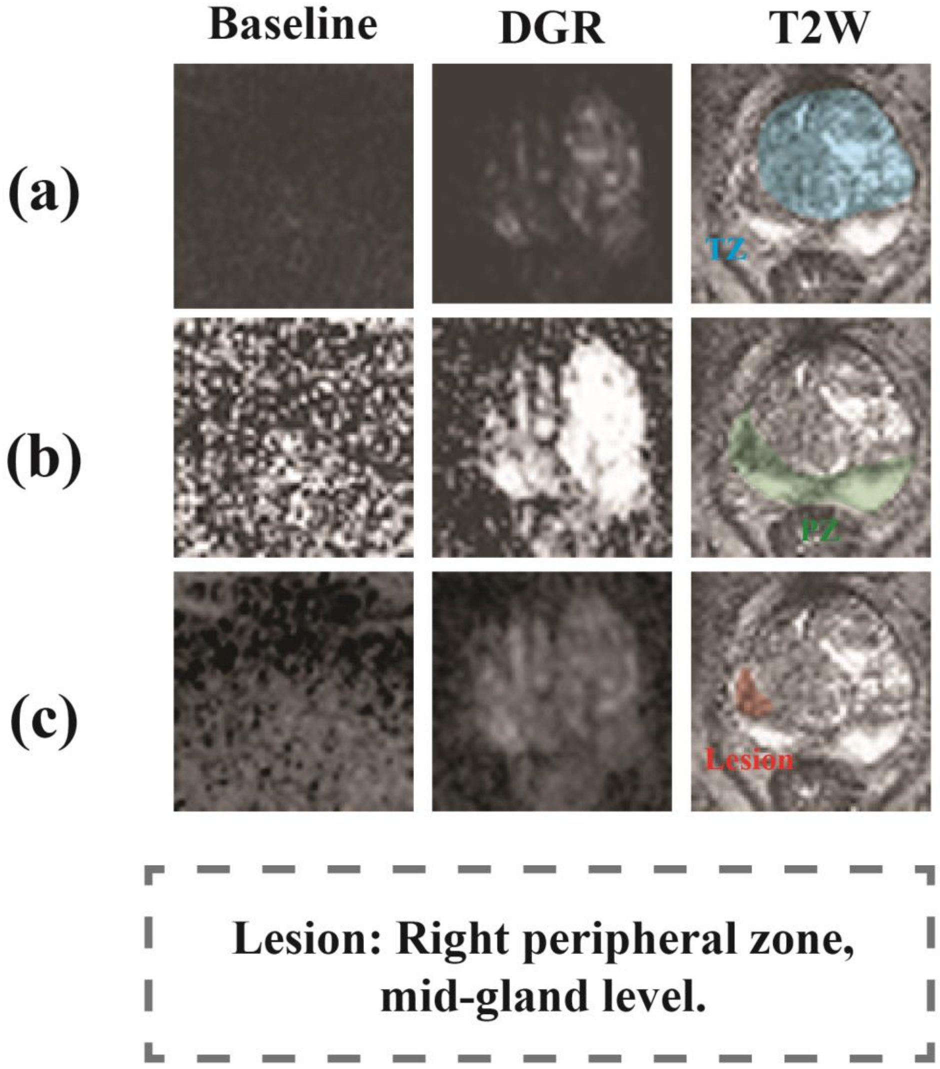

Figure S1. Challenging signal-limited clinical example excluded from the main lesion-related evaluation. Columns show the baseline ssEPI, DGR-corrected image, and co-regridded T2W reference. Rows show (a) low-b DWI (b = 50 s/mm^2), (b) ADC map, and (c) high-b DWI (b = 1400 s/mm^2).

This case illustrates model behavior in a severely signal-limited setting, where the displayed baseline low-b DWI contains little reliable local anatomical information and the restored DWI/ADC remains imperfectly matched to T2W zonal anatomy. Therefore, this example was excluded from the main lesion-related evaluation and should not be interpreted as definitive recovery of the true underlying diffusion signal or quantitative ADC. Instead, it illustrates a boundary condition of the current framework: when local diffusion signal is severely compromised, the restored image may depend more heavily on learned priors, T2W anatomical conditioning, and potentially broader spatial context. Future work should evaluate uncertainty estimation, explicit failure detection, and restoration strategies that incorporate adjacent-slice or fully 3D contextual information.
